# Habitat suitability does not capture the essence of animal-defined corridors

**DOI:** 10.1186/s40462-018-0136-2

**Published:** 2018-09-27

**Authors:** Anne K. Scharf, Jerrold L. Belant, Dean E. Beyer, Martin Wikelski, Kamran Safi

**Affiliations:** 1 Department of Migration and Immuno-ecology, Max Planck Institute for Ornithology, Radolfzell, Germany; 2 Department of Biology, University of Konstanz, Constance, Germany; 3 Camp Fire Program in Wildlife Conservation, State University of New York College of Environmental Science and Forestry, Syracuse, New York USA; 4 Michigan Department of Natural Resources, Marquette, MI USA

**Keywords:** Connectivity, Carnivore conservation, Step selection function

## Abstract

**Background:**

Increases in landscape connectivity can improve a species’ ability to cope with habitat fragmentation and degradation. Wildlife corridors increase landscape connectivity and it is therefore important to identify and maintain them. Currently, corridors are mostly identified using methods that rely on generic habitat suitability measures. One important and widely held assumption is that corridors represent swaths of suitable habitat connecting larger patches of suitable habitat in an otherwise unsuitable environment. Using high-resolution GPS data of four large carnivore species, we identified corridors based on animal movement behavior within each individual’s home range and quantified the spatial overlap of these corridors. We thus tested whether corridors were in fact spatial bottle necks in habitat suitability surrounded by unsuitable habitat, and if they could be characterized by their coarse-scale environmental composition.

**Results:**

We found that most individuals used corridors within their home ranges and that several corridors were used simultaneously by individuals of the same species, but also by individuals of different species. When we compared the predicted habitat suitability of corridors and their immediate surrounding area we found, however, no differences.

**Conclusions:**

We could not find a direct correspondence between corridors chosen and used by wildlife on the one hand, and a priori habitat suitability measurements on the other hand. This leads us to speculate that identifying corridors relying on typically-used habitat suitability methods alone may misplace corridors at the level of space use within an individual’s home range. We suggest future studies to rely more on movement data to directly identify wildlife corridors based on the observed behavior of the animals.

**Electronic supplementary material:**

The online version of this article (10.1186/s40462-018-0136-2) contains supplementary material, which is available to authorized users.

## Background

Changes in land use are affecting many species worldwide through fragmentation and loss of their habitats [1, 2]. Consequently, the affected animals live in an environment where patches of high quality habitat are scattered throughout the landscape. The connectivity between these resulting resource patches depends on the degree to which the landscape facilitates or impedes movement between them [3]. Greater landscape connectivity increases an individual’s ability to cope with many changes in the environment [4, 5]. One way to increase and maintain landscape connectivity is through wildlife corridors [6]. It is therefore important to identify corridors and facilitate their use [7].

Although the corridor concept is intrinsically linked to animal movement [6, 8–10], currently wildlife corridors are generally identified at the population level, relying on habitat suitability measures only. Most studies aim to identify wildlife corridors without a priori knowledge about what a corridor actually is and where they are. The general assumption underlying the prediction of possible corridor locations is that there is a constant habitat preference during all life stages and across behaviors of animal species, although it is known not to be necessarily true [11]. The most widely used method for corridor identification is through an estimation of landscape resistance to movement [12]. In these landscape resistance models the permeability of the landscape to movement is determined by using the inverse of the habitat suitability as a resistance surface. Some studies have included movement data in their habitat models [9, 10], but ultimately they all identify corridors based on habitat properties. The corridors identified through landscape resistance models, tend to be mostly swaths of habitat with higher suitability embedded in a matrix of habitat with lower suitability [13]. As these methods do not treat the corridors as independent units, it is possible that characteristics of habitat that determine corridors above and beyond habitat suitability are neglected. These same methods have been used to identify corridors across different scales, e.g. connecting areas 100–1000 km apart (e.g. [14–16]), or at smaller scales connecting areas 10–50 km apart (e.g. [9, 10, 17]). Although it could be reasonable to assume ecologically that the factors driving corridor use are the same across scales, they could be scale dependent [18]. Within home range corridor use happens at a third-order selection (*senu* [19]), which will be constrained by the second-order selection for the home range placement in the landscape. If the second-order selection is strong, one might expect the third-order selection to be random, hence the movement corridors are independent from habitat suitability, and more movement driven. On the contrary, if second-order selection is rather weak, one might expect a stronger third-order selection, and movement corridors are more habitat driven. LaPoint and colleagues [6] developed an algorithm to identify wildlife corridors solely based on movement, identifying those areas where the animals show quick and parallel movement behavior. In this study they analyzed the corridor behavior of one species (*Pekania pennanti*) and detected, using camera traps, that these areas were also used more often than random by other species within the study area. To understand where corridors occur, and what shapes them, we need to understand the drivers of this corridor behavior.

We identify corridors within home ranges of 60 individuals of four large carnivore species, relying exclusively on their movement characteristics. Thus, we identify corridors independently of environmental features, to investigate the theoretical assumption of a relationship between corridors and habitat suitability. Corridors are mostly identified at a larger scale, aiming to connect populations and communities [15], but individuals not only rely on corridors during migration, seasonal home range shifts [17] or during dispersal [14], but also within their home ranges, especially when living in fragmented landscapes. Corridors are important at all scales, and few studies [6, 9] have evaluated corridors at the individual home range level. We predict that individuals with home ranges containing more heterogeneous landscape use corridors more often, as a greater heterogeneity of the landscape could imply greater patchiness of suitable habitat to be connected through corridors within their home ranges. We test whether the corridors within home ranges too are swaths of suitable habitat surrounded by less favorable habitat as shown for corridors at larger scales. Finally, we test whether corridors have an environmental composition that consistently differs from the environmental composition of the home range, which in turn would give us a better understanding of potential drivers shaping corridors at a home range level and enable us to better predict them spatially*.*

## Methods

### Study area

**Fig. 1:**
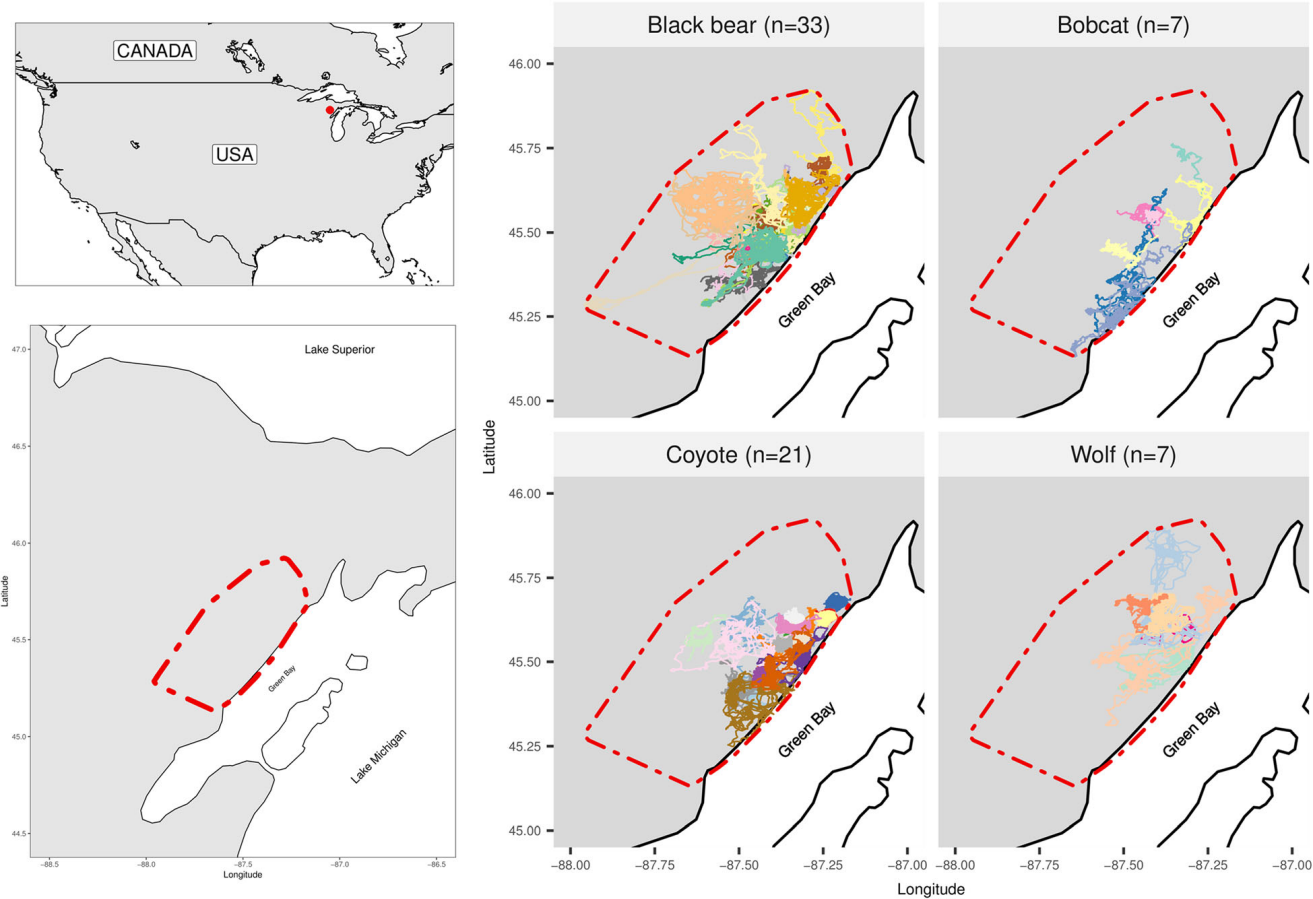
Study site. Red polygons represent the 100% MCP (minimum convex polygon) containing all individuals of all species. Left panel: colored lines represent the tracks of the different individuals. “n” represents the number of tracks

The study area covered about 2800 km^2^ within Delta and Menominee counties in the Upper Peninsula of Michigan, USA (45°35′0.00"N, 87°23′0.00"W, Fig. 1). The main land cover types of the study area included woody wetlands (44%) (e.g., black spruce *Picea mariana*, green ash *Fraxinus pennsylvanica*, northern white cedar *Thuja occidentalis*, speckled alder *Alnus incana*), deciduous forest (17%) (e.g., sugar maple *Acer saccharum*, quaking aspen *Populus tremuloides*), and agriculture (12%) (i.e., row crops and pastures). The remaining 27% of the study site included conifer forest, mixed forest, urban areas, roads, herbaceous wetlands, shrub, and open water [20, 21]⁠. Land covers with direct human influence (agriculture, urban areas and roads), represented 18% of the study area. The area is relatively flat, with an elevation range of 170 to 310 m.a.s.l. and mean road density of 1.2 km/km^2^.

### Tracking data

During March–August 2009–2011 we captured and immobilized 25 black bears (*Ursus americanus*), 7 bobcats (*Lynx rufus*), 21 coyotes (*Canis latrans*) and 7 wolves (*Canis lupus*) (Table 1). We fitted all individuals with Lotek GPS collars (model 7000MU for black bears and 7000SU for bobcats, coyote and wolves; Lotek Wireless, Newmarket, Ontario, Canada), programmed to obtain a location every 15 min between the 1 May and 30 September. We located black bears collared the previous year in their winter dens, immobilized them and replaced their GPS collars. The collars fitted to bobcats, coyote and wolves included drop-off mechanism to release collars 30 weeks after deployment. Location data from all collars could be downloaded remotely. For the data analyses in this study we used GPS locations recorded from 1 May - 30 September 2009–2011. If individuals were trapped during this time window, we removed the first 5 days of tracking data collected after collaring to avoid possible effects of capture and handling on movements and habitat selection analysis. For the 8 black bears that were monitored over consecutive years, we analyzed data from each year separately. For each individual we calculated the range distribution, as the 100% minimum convex polygon, which represents the broad space required by the animal, i.e. the home range. We also calculated the occurrence distribution, which estimates where the animal was located during the observation period [22]. We calculated the occurrence distributions with the dynamic Brownian bridge movement model [23] with the R package *move* [24] (Additional file 1).

**Table 1:** Summary of individuals and tracks included in study

Species	Number of individuals	Sex		Life stage		Number of days tracked^a^ (mean ± SD)	Number of locations^a^ (mean ± SD)
		Female	Male	Adult	Juvenile		
Black bear	25 (+ 8)^b^	10 (3)^b^	14 (5)^b^	20 (8)^b^	5	82 ± 37	7431 ± 3511
Bobcat	7	1	6	5	2	87 ± 44	8166 ± 4090
Coyote	21	11	10	19	2	101 ± 29	9435 ± 2762
Wolf	7	5	2	7	0	105 ± 26	9504 ± 2298

^a^Only the data used in the analysis of this study is included (i.e. within the time window of interest,1st May - 30th September)

^b^Number of individuals that were tracked in consecutive years for which tracks of different years were analyzed separately

### Environmental data

We obtained land cover data from the 2011 National Land Cover Database [21] at 30 m resolution, and complemented this map with data for highways, secondary roads [25], rivers and lakes [26]. We rasterized highways, secondary roads, rivers and lakes at the resolution of the land cover data (30 × 30 m). Though most roads and rivers are not 30 m wide, our GPS collars had a position error of about 20 m and we considered this resolution adequate for our analysis. We reclassified the pixels in the original land cover layer corresponding to highways, secondary roads, rivers and lakes (Additional file 2). Finally we reclassified land cover data into 7 land cover classes (human development, open cover, evergreen forest, mixed forest, deciduous forest and woody wetland), and calculated for each 30 m grid cell the percentage of each land cover type within a 30 m radius around it. We also calculated the distance from the centroid of each grid cell to water, highways and secondary roads. We excluded those areas classified as lakes for analyses of habitat suitability. We did not include topography, as the study area had low topographic relief.

### Corridors

We used the *corridor* function in the R package *move* [24] to locate the animal defined corridors as described by LaPoint and colleagues [6]. We used the function’s default settings, selecting the upper 25% of the speeds (speedProp = 0.75), and lower 25% of the circular variances of the pseudo-azimuths of the segments midpoints, measurement used to identify the near parallel segments (circProp = 0.25). This method identifies wildlife corridors relying solely on characteristics of animal movement behavior, classifying the locations of a track into corridor and non-corridor. We calculated for each individual the occurrence distribution of the locations classified as corridors. We defined each contiguous area of the 95% occurrence distribution as a corridor polygon. We identified those corridor polygons composed of only three or less consecutive locations as “outlier” corridors, and reclassified these locations as non-corridor (Additional file 3). After reclassifying the outliers, we calculated two separate occurrence distributions per individual, one for the corridor locations, and one for the non-corridor locations. From the obtained occurrence distribution for the corridor locations, we defined each contiguous area of the 95% occurrence distribution as a corridor polygon, and calculated the maximum length and average width of these polygons with the R library *lakemorpho* [27]. To test whether the areas around the corridors had lower habitat suitability, we identified the area immediately surrounding each corridor polygon (Additional files 1 and 4), with a different width for each species. The width corresponded to the species average step length between locations identified with corridor behavior (black bear: 300 m; bobcat: 200 m; coyote: 400 m; wolf: 600 m).

Once corridors were identified, we investigated if the same corridor was used by the same individual in different years, by individuals of the same species in the same year and in different years, and by individuals of different species in the same and different years. We did this by calculating the degree of spatial overlap of all corridor polygons. We superimposed all the corridor polygons and calculated the percentage of overlap for each of the overlapping polygons. We counted each overlapping pair once, always the one with the highest percentage of overlap.

### Landscape heterogeneity

We used the Hill numbers diversity index to measure landscape heterogeneity [28]. The Hill numbers, a modified Shannon Index, takes into account that the number of land cover types present in each home range is different. This enabled us to compare the diversity indices derived from home ranges with different number of land cover types. We extracted for each individual the number of pixels of each land cover type (Additional file 2) within its home range. We used these frequencies to calculate the Shannon Index for each individual home range using the function *diversity* of the R package *vegan* [29]. The Hill numbers index is obtained by calculating the exponential of the Shannon Index. The higher the Hill numbers index, the higher the diversity in land cover types within the individuals’ home range, which indicates a higher heterogeneity of the landscape. We also calculated the Hill numbers index of the corridor polygons and the 95% occurrence distribution of the non-corridor locations of each individual, to test if there were differences in landscape heterogeneity among the home range and the occurrence distribution, i.e., where the animal was observed, differentiating between corridors and non-corridors. We compared the diversity indices of these three areas by means of three pairwise t-tests per species.

We considered two variables as indicators of intensity in corridor use. First, we considered the number of corridor segments identified within an individuals’ track, second, we accounted for the number of corridor polygons present in the home range. To investigate if the landscape heterogeneity was determining the intensity in corridor use, we fit one generalized linear model (GLM) with a Poisson distribution, where the number of corridor segments per individual was our dependent variable, and the Hill numbers index, the home range size (m^2^) and the number of days the individual was tracked were included as explanatory variables. And we fit another GLM with number of corridor polygons as a dependent variable, and the same explanatory variables as in the previous model. We fitted both models for each species separately, because the sample sizes were very different between species. We also calculated the Pearson’s correlation coefficient between the number of corridor segments and the number of corridor polygons per species.

### Habitat suitability

We calculated habitat suitability using a step selection function (SSF, [30]). This function compares the environmental attributes of an observed step (based on two consecutive GPS locations) with a number of random steps that have the same starting point. As observed steps we included those steps with a time lag of approx. 15 min, excluding steps with missing fixes. We generated the random steps from a multivariate normal distribution, using the function *rmvnorm* of the R package *mvtnorm* [31], maintaining the variance/covariance structure of speed and turning angle of the empirical track of each individual. The variance/covariance structure of speed and turning angles used to create these random steps was based on the steps without missed fixes. We used 5 random steps per observed step, converting speed to step length by multiplying the random speed by the time between fixes of the corresponding observed step. To model habitat suitability, we compared the environmental characteristics of the end points of each observed step with its 5 corresponding random steps in a binary conditional logistic regression model using the *clogit* function of the R package *survival* [32]. The explanatory variables included the proportion within a 30 m radius of human cover, open cover, evergreen forest, mixed forest, deciduous forest and woody wetland, distance to roads, and distance to water. We also included step length and relative turning angle as explanatory variables in the model as the likelihood of realizing a specific option is a function of these two measurements. This accommodates persistence in movement and the relationship between speed and turning angle. When animals move, they will be likely to maintain both their direction of movement and speed as well as a certain relationship between the two metrics. When moving fast (i.e. cover larger distances per time unit) they will be moving with low tuning angles, while when turning resulting in a high turning angles, they usually do so while moving slowly (i.e. covering shorter distances per time unit).

We built a series of SSF models to investigate the habitat suitability and the environmental composition of the home ranges and the corridors. We built one model per individual which contained all locations (*full SSF model*) and calculated the habitat suitability prediction within its home range. Each *full SSF model* per individual was based on 75% of the randomly selected observed locations. We used the remaining 25% of the locations for posterior cross-validation. For each individual we calculated the predicted habitat suitability. For each prediction, we kept distance and relative turning angle constant, selecting a random pair of values from the observed locations. To make the results comparable, we rescaled the predicted values between 0 and 1. We rescaled the data using the normalization formula X’ = (X_i_ – X_min_) / (X_max_ – X_min_), where X’ is the rescaled and X_i_ the original value. To evaluate the model performance we extracted the predicted value for the 25% excluded observed locations, and also for the same number of random locations selected from the individual’s home range. We repeated this a 100 times. With a Kolmogorov-Smirnov test we compared the distribution of the predictive values of the observed locations with each set of random locations. We used the predictions from the *full SSF models*, to compared the predicted habitat suitability values between corridor and non-corridor locations, to test if there were differences between them. For this we extracted the habitat suitability value for each location, calculated the mean ± SD for the corridor locations and non-corridor locations per individual and compared these two values by means of a t-test. We also compared the predicted habitat suitability of each corridor polygon with its immediate surrounding area to investigate if the corridors were surrounded by habitat of lower suitability. For this we extracted the mean ± SD habitat suitability values of the corridor polygons and of their immediate surrounding area, and compared these values using a paired t-test.

We built another SSF model per individual this time only including corridor locations (*corridor SSF model)* to find out if corridors could be predicted in space at the individual level*.* We calculated the prediction of this model and assessed how well it predicted the corridor locations compared to random points sampled from the individuals home range. Each *corridor SSF model* per individual was based only on corridor locations. For the calculation of random steps of the *corridor SSF model*, we used the variance/covariance structure of speed and turning angle of the non-corridor steps. We calculated the difference between the mean predictive value of corridor locations and the mean predictive value of random locations sampled in the individual’s home range, to test how well the *corridor SSF model* could predict corridors. For each individual we sampled the same number of random locations as they had corridor locations, and calculated the difference between the means of the model predictions. We repeated this procedure 100 times to obtain a better estimate of the mean differences of model prediction for random versus corridor locations for each individual.

Finally we wanted to test whether the underlying environmental characteristics of corridors and non-corridors differed. One possible simple approach would be to develop a model for each group of locations, and assess the models’ ability to predict the environmental composition of the other group. However for our tracked individuals on average (± SD) only 0.51 ± 0.35% of the total locations were identified as corridors. Notably, any observed difference could be due to differences in sample size rather than reflecting a true difference in the underlying environmental characteristics between these two groups of locations. Therefore, we built for each individual 1000 SSF models including in each of them a random subset of non-corridor locations (*non-corridor SSF model*). Each random subset contained the same number of non-corridor locations as corridor locations of the individual. We evaluated the ability of the *corridor SSF model* and the *non-corridor SSF models* to predict the corridor locations and assessed if the prediction ability differed between these two models. For this we calculated the mean prediction value of the corridor locations for each of the 1000 *non-corridor SSF models* and for the *corridor SSF model*. We then assessed whether the mean predicted value of the *corridor SSF model* was within the distribution of predicted values of the *non-corridor SSF models*. All calculations were done in R 3.3.1 [33].

## Results

### Corridor identification and intensity of corridor use

**Fig. 2:**
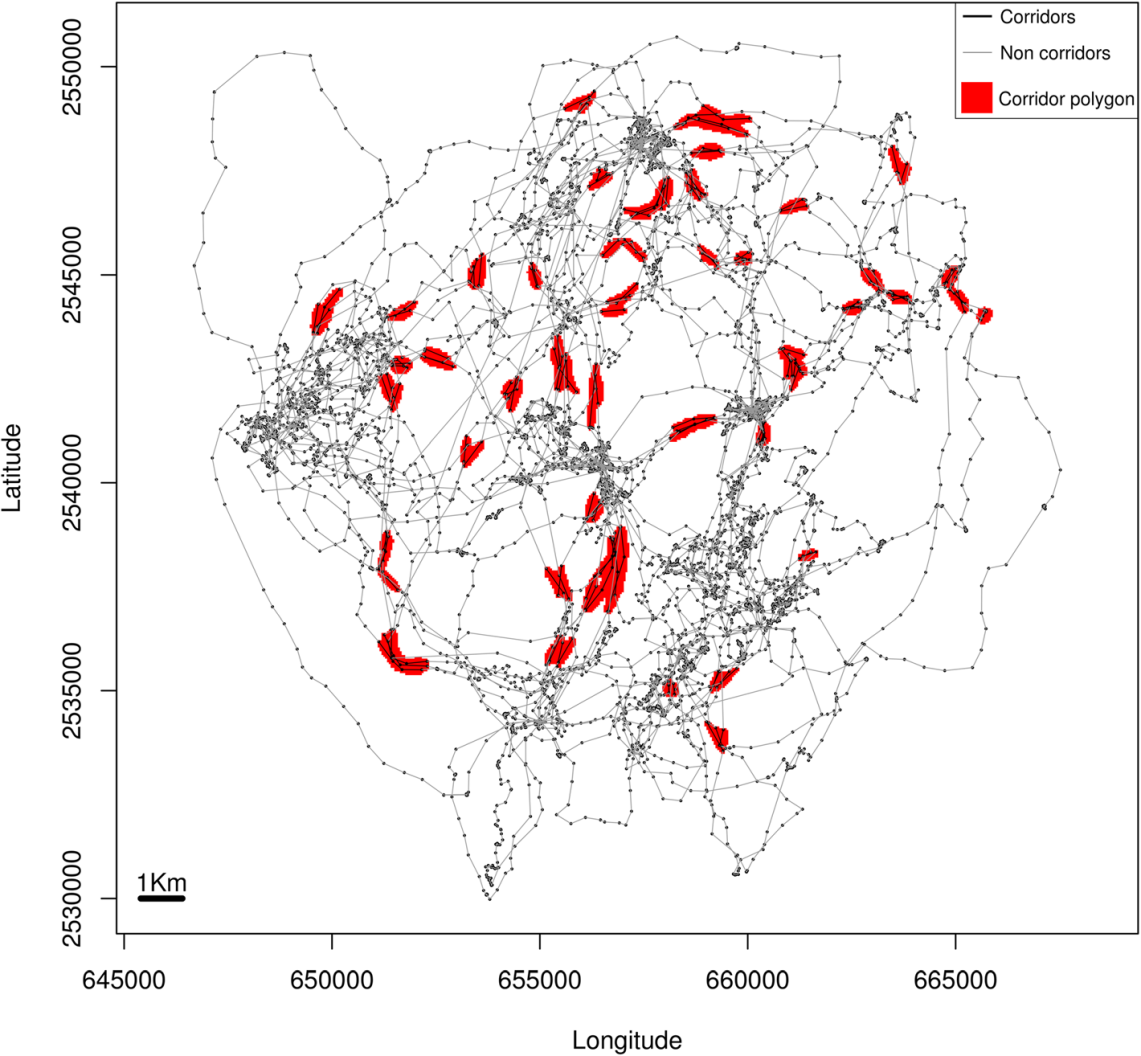
Corridor segments and polygons (Example of one black bear tracked 130 days in 2011)

All tracked individuals (Table 1), except for one black bear, one bobcat and one coyote, showed corridor usage (Fig. 2). The number of corridors each individual presented was highly variable across all individuals of all species. We found an average of 42 ± 34 (mean ± SD) corridor segments (black bear: 48 ± 38, bobcat: 22 ± 20, coyote: 31 ± 16, wolf: 70 ± 41) and of 11 ± 8 corridor polygons across all individuals (black bear: 13 ± 10, bobcat: 7 ± 5, coyote: 8 ± 5, wolf: 13 ± 7). The number of corridor segments were highly positively correlated with corridor polygons (in black bears: *r* = 0.967, DF = 31, *p* > 0.001, bobcats: *r* = 0.918, DF = 5, *p* = 0.003, and coyotes: *r* = 0.773, DF = 19, p > 0.001). In wolves the correlation was also positive, but not significant (*r* = 0.669, DF = 5, *p* = 0.099). With increasing home range size and days of tracking the number of corridor segments identified increased in black bears, coyotes and wolves, but decreased for bobcats. We found that with increasing landscape heterogeneity the number of corridor segments increased for black bears and decreased for wolves. For bobcats and coyotes we did not find a significant relationship between the number of corridor segments and landscape heterogeneity. We found similar relationships between the number of corridor polygons and home range size, number of days tracked and landscape heterogeneity (Table 2).

**Table 2:** Results of generalized linear models explaining intensity of corridor use

Species	Variable	Corridor segments		Corridor polygons	
		ß (*p*-value)	Deviance explained	ß (*p*-value)	Deviance explained
Black bear	Landscape heterogeneity (Hill numbers)	0.1168 (**)	69.83	0.1172 (ns)	77.83
	Home range size (m^2^)	0.0014 (***)		0.0014 (***)	
	Number of days tracked	0.0150 (***)		0.0156 (***)	
	Intercept	1.5912 (***)		0.2156 (ns)	
Bobcat	Landscape heterogeneity (Hill numbers)	−0.0347 (ns)	84.07	−0.0159 (ns)	71.74
	Home range size (m^2^)	−0.0020 (**)		−0.0003 (ns)	
	Number of days tracked	0.0241 (***)		0.0165 (***)	
	Intercept	1.1553 (*)		0.4291 (ns)	
Coyote	Landscape heterogeneity (Hill numbers)	0.0470 (ns)	24.87	0.0571 (ns)	44.08
	Home range size (m^2^)	0.0017 (***)		0.0029 (***)	
	Number of days tracked	0.0060 (***)		0.0017 (ns)	
	Intercept	2.3062 (***)		1.1291 (*)	
Wolf	Landscape heterogeneity (Hill numbers)	−0.5444 (***)	49.80	−0.3981 (*)	83.32
	Home range size (m^2^)	0.0012 (***)		0.0017 (***)	
	Number of days tracked	0.0037 (ns)		−0.0019 (ns)	
	Intercept	6.5771 (***)		4.4767 (***)	

Signif. codes: 0‘***’ 0.001 ‘**’0.01 ‘*’0.05 ‘.’ 0.1 ‘ns’ 1

Corridors varied in size across species; the shortest (148 × 79 m) was from a bobcat and the narrowest (174 × 51 m) from a black bear, while the longest (7878 × 941 m) and widest (3189 × 1572 m) corridors were from wolves. Wolves had on average (± SD) the longest corridors (1385 ± 998 m) and bobcats the shortest (372 ± 157 m). Black bears and coyotes had a similar mean corridor size of 727 ± 406 m and 792 ± 424 m, respectively. The mean aspect ratio of the corridors for all species was similar with a mean (± SD) of 2.8 ± 1.1 (range = 1.2–10.9).

**Fig. 3:**
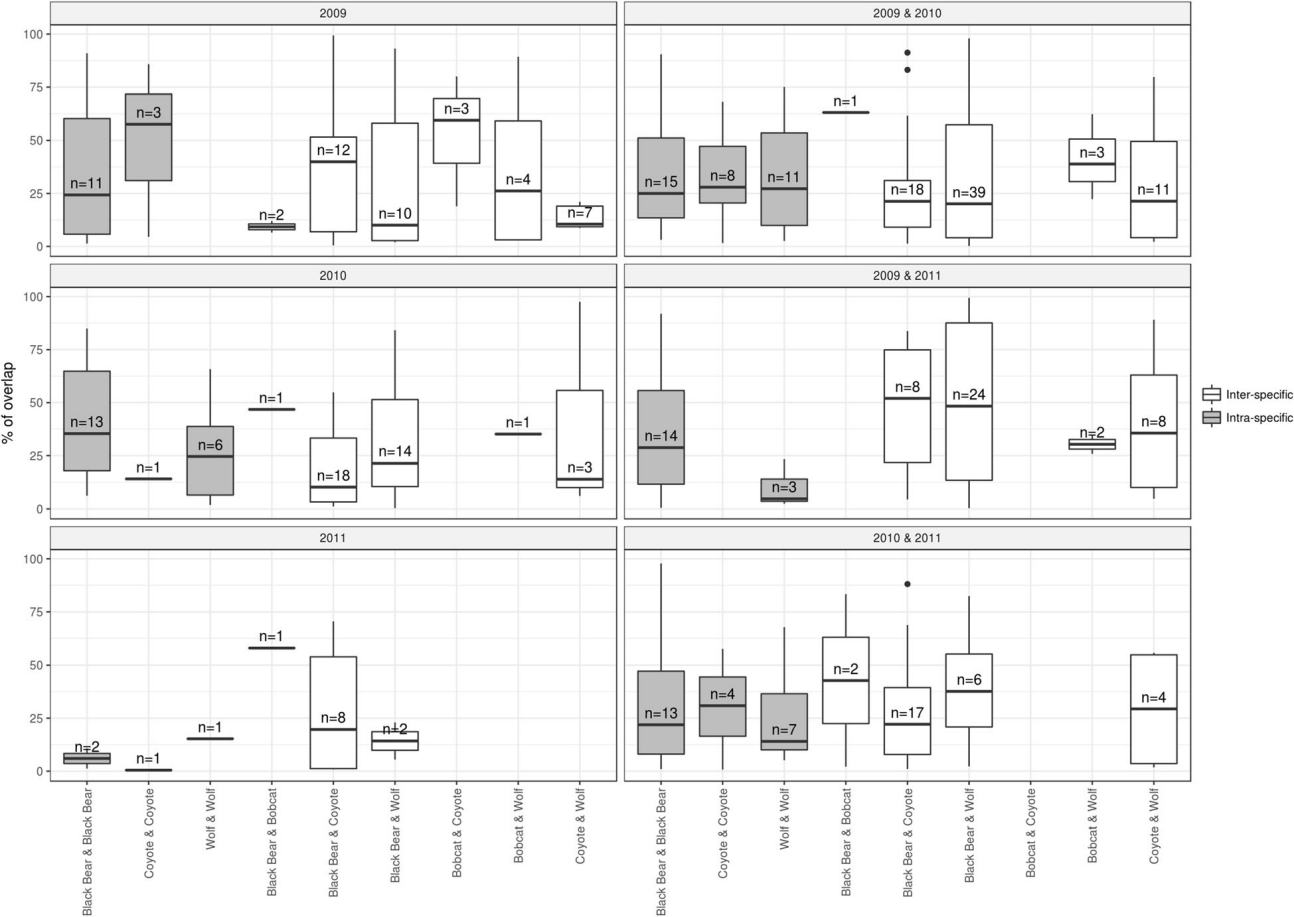
Overlap of corridors. Percentage of overlap of corridors within species and among species within the same year and among years. Each overlapping pair is counted once, always the one with the highest percentage of overlap. “n” represents the number of overlapping pairs of corridors. Overlap between corridors used by the same individual over several years are excluded, and represented separately in Additional file 5

**Fig. 4:**
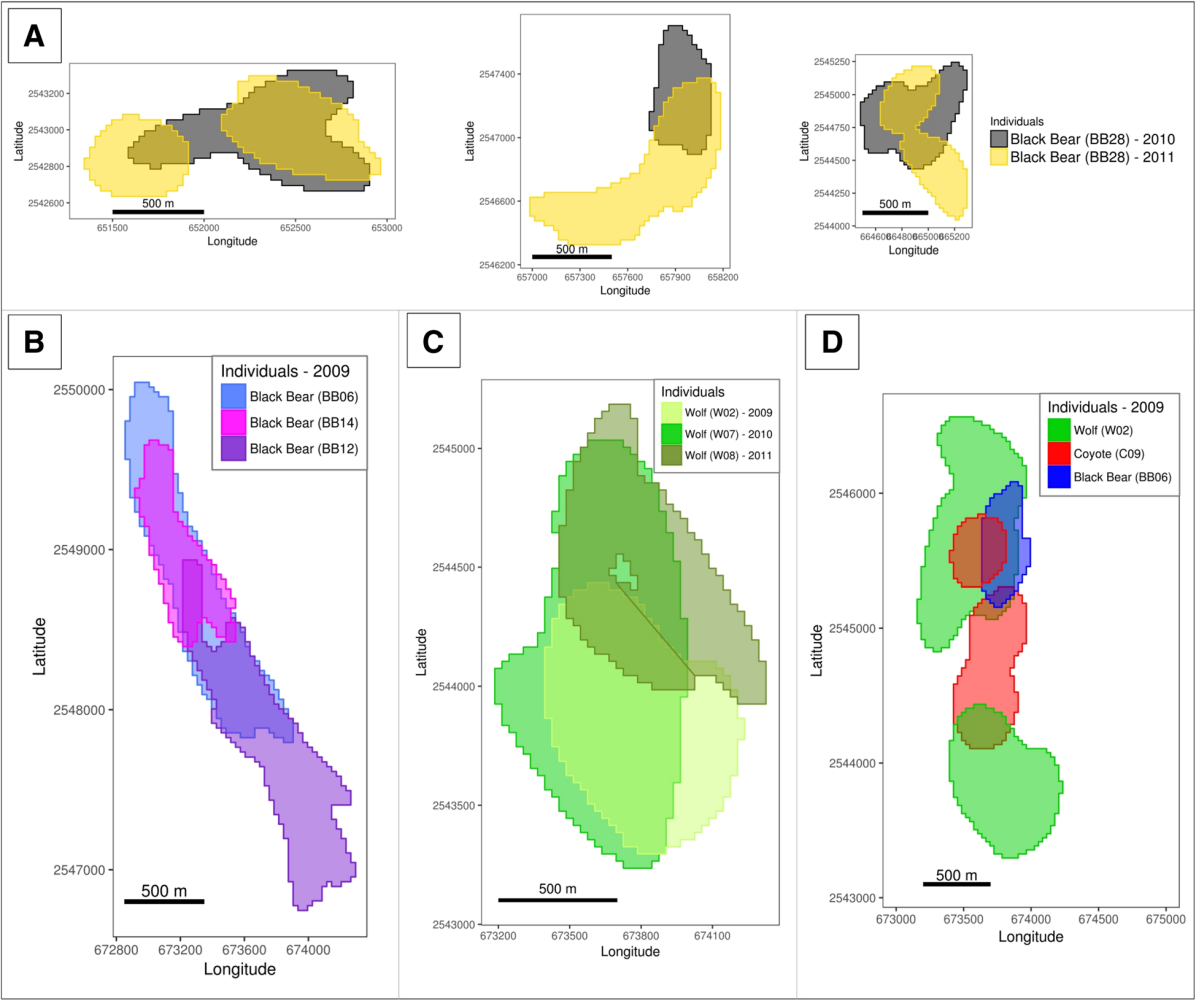
Examples of overlaying corridors. (**a**) Corridors of 2 different years of the same black bear. (**b**) Corridors of 3 black bears overlapping in the same year. (**c**) Corridors of 3 wolves overlapping in different years. (**d**) Corridors of 3 individuals of 3 species overlapping in the same year

We found corridors to be used by the same individual over several years, and also corridors used by several individuals of the same or different species. Black bears showed the highest number of corridors shared intra-specifically, but they also had the highest overlap with all other species, especially coyotes and wolves. In contrast, bobcats did not share corridors intra-specifically, and their corridors only occasionally overlapped with those of other species (Fig. 3, Additional file 5). We found all possible combinations of overlapping corridors including a black bear that used several of the same corridors during the 2 consecutive years (Fig. 4a), overlapping corridors of individuals of the same species tracked during the same (Fig. 4b), or different years (Fig. 4c), and overlapping corridors of individuals of different species tracked in the same year (Fig. 4d).

### Habitat suitability within and around corridors

The *full SSF model* performed well in predicting overall habitat suitability (Additional file 6). The distribution of the predicted values of habitat suitability of the locations left out for validation, differed from the distribution of randomly sampled locations from the prediction map for all individuals (Kolmogorov-Smirnov tests; black bear: D = 0.30 ± 0.12, *p* < 0.001; bobcat: D = 0.25 ± 0.10, p < 0.001; coyote: D = 0.28 ± 0.10, p < 0.001; wolf: D = 0.32 ± 0.18, p < 0.001; D = mean ± SD). Habitat suitability was lower in corridors than in non-corridors for the vast majority of individuals across all species (black bear: 91%, bobcat: 86%, coyote: 95%, wolf: 100%). Although for 75% of these individuals this difference was significant, the difference between the values was very small, 0.05 ± 0.03 (mean ± SD across all individuals and species, Additional file 7). Interestingly, we did not find a significant difference between habitat suitability within the corridor polygon and its immediate surrounding area (Additional file 8).

### Environmental composition of corridors

**Fig. 5:**
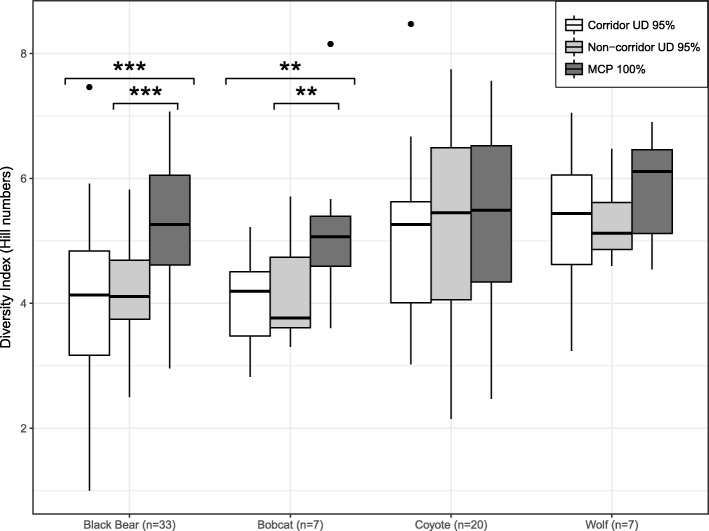
Landscape heterogeneity comparison in home range, non-corridor occurrence distribution and corridor occurrence distribution. Significance codes: < 0.001***, < 0.01** . All other pairs did not show a significant difference. “n” represents the number of individuals included in the analysis

For black bears, bobcats and wolves the landscape heterogeneity within the corridor and non-corridor areas was lower than within the entire home range. For wolves this difference was not significant. We did not find any differences in landscape heterogeneity for coyotes (Fig. 5). When comparing the landscape heterogeneity between corridor and non-corridor areas we did not find any differences in any of the species (Fig. 5).

The *corridor SSF models* which we built to predict corridors in space had a poor performance. Although for most individuals these models were able to predict the corridor locations better than random locations drawn from their home range (Additional file 9), the differences between the predictive values of the corridor and the random locations were very small, 0.037 ± 0.031 (mean ± SD across all tracks with corridor behavior (*n* = 67)). We also found that the underlying environmental characteristics between corridors and non-corridors was not distinguishable for most individuals. Only in 12% of the individuals on average (black bears: 15%, bobcats: 17%, coyotes: 10%, wolves: 0%), the predictions of corridor locations from the *corridor SSF model* were better than 95% of the values obtained from the *non-corridor SSF models* (Additional file 10).

## Discussion

We found that corridors identified solely by the movement behavior of the individuals within a home range, were virtually indistinguishable from the surrounding areas using typical landscape metrics previously employed in corridor studies. Our corridor models predicted corridor locations marginally better than random points. As the corridor models were based on locations that also correspond to a behavioral state of traveling, they probably predicted areas suitable not only for corridors, but for all directed movements [9]. Additionally, the sample size of relocations used to build the corridor models was quite small, which could have influenced the model performance.

We observed a high degree of variation in the number of corridors used by individuals tracked in our study. This variation was partly explained by the duration of the tracking period. The longer an individual was tracked increased the chances of recording revisits to the same areas and thus detecting corridor behavior. Beyond tracking duration, however, landscape heterogeneity could not completely explain the remaining variation. We expected a greater heterogeneity of the landscape to imply greater patchiness of the habitat, which seemed not necessarily to be the case. LaPoint and colleagues [6] found a difference in corridor use between fishers (*Pekania pennanti*) occupying home ranges with different levels of landscape heterogeneity. In their study fishers located in a heterogeneous suburban area showed corridor usage, whereas those inhabiting a homogeneous forested area did not use corridors. The landscape heterogeneity among the tracked individuals in our study may have been less extreme than in previous studies, wherefore we might not have been finding a clear relationship between the variation in intensity of corridor use and the landscape heterogeneity.

The areas identified as corridors in most individuals across all species had lower habitat suitability values than the areas identified as non-corridors. However, the small differences detected can hardly be considered biologically relevant. The variability of habitat suitability was both within corridor and non-corridor location an order of magnitude higher than the difference between these 2 groups of locations. The corridors also did not contain habitat with higher suitability than the adjacent areas surrounding them. These findings clearly fail to support the general theoretical assumption of corridors being defined as relatively suitable habitat surrounded by less suitable habitat (e.g. [12, 13, 34]) providing the basis for a resistance landscape. Nevertheless, we cannot exclude the possibility that the choice of the size of the immediate surrounding area of the corridor might have been too large. By including areas that should have been considered non-corridors, we might have missed potential differences. The size of the area delineating a corridor likely depends on many factors. For example, it could depend on the perception distance of the species, the characteristics of the landscape or the length of the corridor and there may not be a means to define corridors generally. We chose an average step length distance as the width of the immediate surrounding area, because in the multiple times they used the corridor, theoretically they could have, at any time, taken a step outside of the corridor instead of continuing straight ahead. We assumed this directed movement shaping the corridor to be an “avoidance” behavior to the surrounding area of the corridor.

Where corridors come to be placed in the landscape is probably a consequence of many factors. Their placement could depend on environmental features that are not detected with existing remote sensing technology or our analysis, such as the permeability of the landscape (e.g. vegetation density of the forest understory). Individuals likely select their travel paths where the composition of the vegetation provides the least physical resistance to movement. It is possible that land cover itself is not the most important factor, but the geometry of the patch itself or that of the neighboring patches. Furthermore, the spatial location of corridors in the landscape can be also “learned” and inherited and thereby have become landscape features themselves. Individuals of the same pack or family group may “learn” a given path from the other members of the group, and reuse this path, e.g. a convenient place to cross a road or river [35]. Our results demonstrate spatial overlap of corridors from multiple individuals of the same and different species. Although these findings are anecdotal, as only a very small portion of the animals present in the study area were captured and tagged, they also represent minimum estimates of corridor use by multiple individuals. Often a diversity of species are recorded along corridors identified for one individual (e.g. [6]). This result supports the idea that a particular feature of those areas rather than the environmental conditions pertaining to specific species’ ecology triggered individuals to exhibit corridor behavior. This finding suggests that identifying corridors used by multiple species simultaneously would ultimately enhance conservation efforts.

We found that at a level of home range movement, animals did use corridors. Our results however suggest that corridors were not directly linked to habitat suitability, and we thus could not identify landscape attributes characterizing them. These results open up the question a as to whether studies that identify corridors using a cost-based model relying on general habitat suitability may place corridors in the wrong places, at least at an individual level within home ranges. The areas where animals of various species chose to establish their corridors, were not the same areas we would have suggested using models relying on habitat suitability models and the set of generally available remote sensing information [6]. We suggest future studies to rely more on movement data when attempting to identify wildlife corridors.

## Conclusions

Surprisingly, most individuals used corridors within their home ranges. Several corridors were used simultaneously by individuals of the same species, but some were also shared between different species. This gives an indication that there probably is something in the environment that triggers the corridor behavior. However, we found no direct link between corridors and habitat suitability, or defining environmental characteristics identifying actual corridors. We also found no difference between predicted habitat suitability of corridors and their immediate surrounding area. This leads us to speculate that identifying corridors relying on the habitat suitability methods only, may misplace corridors at the level of space use within an individual’s home range. We suggest future studies when possible to rely more on movement data than on habitat suitability measures to identify wildlife corridors based on empirical evidence.

## Additional files

Additional file 1: Example of a brown bear’s (BB06, tracked 79 days in 2009) home range, occurrence distributions (for corridor and non-corridor locations) and the immediate surrounding areas of the corridors. (PDF 498 kb)

Additional file 2: Environmental variables used. (PDF 27 kb)

Additional file 3: Corridor outliers calculation. Left panel: track of one wolf (W01), with all corridors identified by the corridor function of the *move* R package. Right panel: detail of one section of the track. Corridors A, B and C are considered as outliers. Corridor D would be accepted as corridor. (PDF 110 kb)

Additional file 4: Detail of corridor polygons and their immediate surrounding areas. (PDF 257 kb)

Additional file 5: Overlap of corridors of the same individual. Percentage of overlap of corridors within one black bear tracked over several years. Each overlapping pair is counted once, always the one with the highest percentage of overlap. “n” represents the number of overlapping pairs of corridors. (PDF 183 kb)

Additional file 6: Example of *full SSF model* and *corridor SSF model* predictions for one black bear, bobcat, coyote and wolf. Prediction area corresponds to the individuals’ home range. (PDF 4305 kb)

Additional file 7: T-test results of the comparison between the habitat suitability value (HS) of corridor vs non-corridor locations from the *full SSF model.* Negative value of “t” implies that the corridor locations had lower habitat suitability than the non-corridor locations. (PDF 36 kb)

Additional file 8: Paired t-test results of the comparison between the mean habitat suitability value of the corridor polygon and its immediate surrounding area from the *full SSF model.* Negative value of “t” and “mean of differences” imply that the corridor polygon had lower habitat suitability than the immediate surrounding area. (PDF 33 kb)

Additional file 9: Prediction success of *corridor SSF models*. Dark gray: individuals where corridor locations had higher prediction value than random locations. Light gray: individuals where random locations had higher prediction value than corridor locations. (PDF 29 kb)

Additional file 10: Comparison between prediction of corridor locations by the *corridor SSF model* and the *non-corridor SSF models.* The black line represents mean prediction value of the *corridor SSF model*, and the colored area represents the distribution of the mean predictions of the 1000 repetitions of the *non-corridor SSF models.* When the line is to the right of the largest peak of the distribution of the predictions of the *non-corridor SSF models*, the *corridor SSF model* could predict better the corridor locations (e.g. BB05, BC07, etc). In all other cases the *non-corridor SSF model* could predict the corridor locations as good or better than the *corridor SSF model*. Red: black bears; yellow: bobcats; dark blue: coyotes; light blue: wolves. (PDF 246 kb)

## Abbreviations

GLM: Generalized linear model
GPS: Global positioning system
km: Kilometers
m: Meters
MCP: Minimum convex polygon
SD: Standard deviation
SSF: Step selection function
