# Supplementary material for: Habitat suitability does not capture the essence of animal-defined corridors
Source: Mov Ecol. 2018 Sep 27;6:18. doi: 10.1186/s40462-018-0136-2 (PMC6158861; doi:10.1186/s40462-018-0136-2)
Supplement: Supplementary file 1 — Example of a brown bear’s (BB06, tracked 79 days in 2009) home range, occurrence distributions (for corridor and non-corridor locations) and the immediate surrounding areas of the corridors. (PDF 498 kb) [file 40462_2018_136_MOESM1_ESM.pdf]

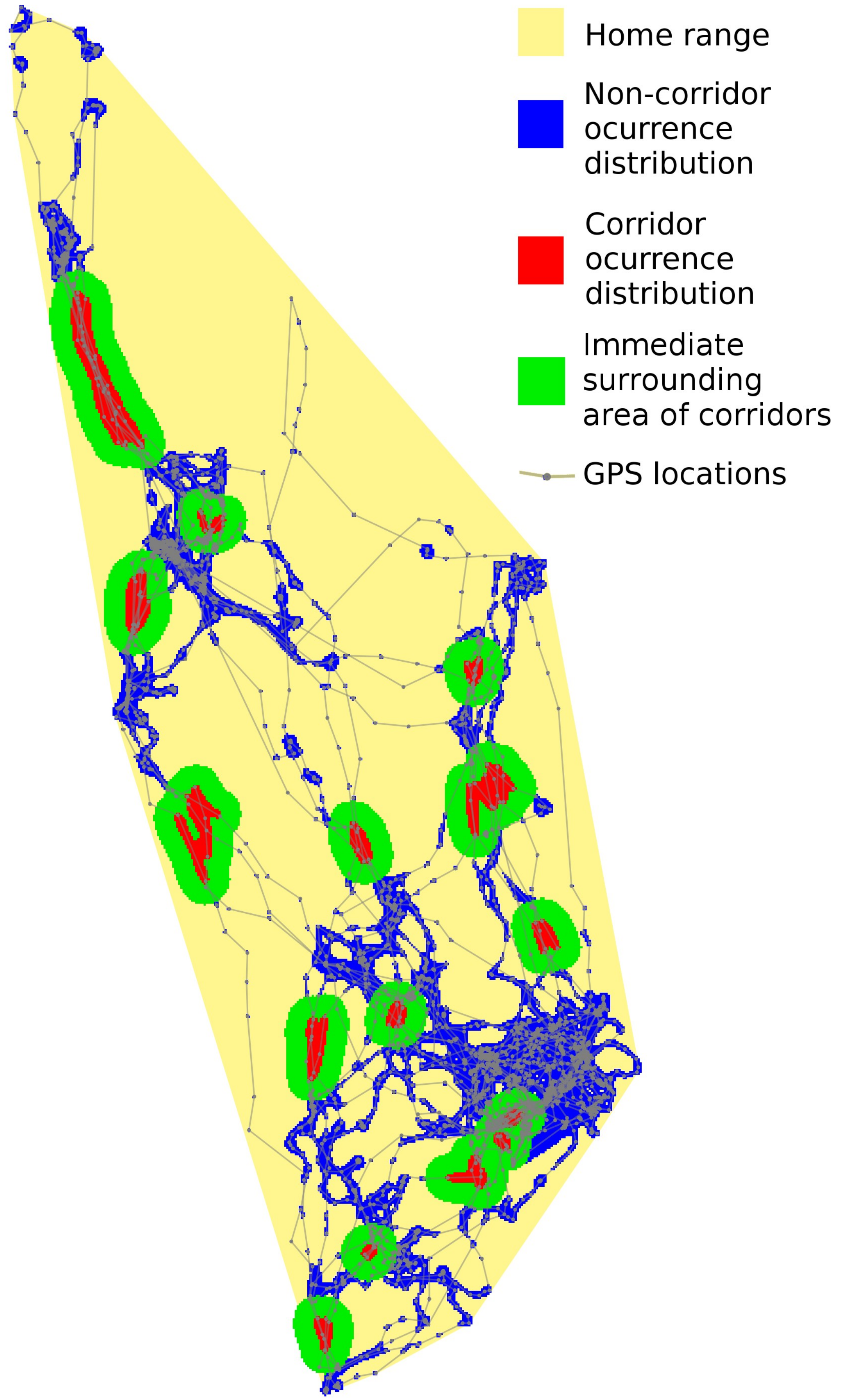


**Additional file 1. Example of a brown bear's (BB06, tracked 79 days in 2009) home range, occurrence distributions (for corridor and non-corridor locations) and the immediate surrounding areas of the corridors.**
