# Supplementary material for: Habitat suitability does not capture the essence of animal-defined corridors
Source: Mov Ecol. 2018 Sep 27;6:18. doi: 10.1186/s40462-018-0136-2 (PMC6158861; doi:10.1186/s40462-018-0136-2)
Supplement: Supplementary file 2 — Environmental variables used. (PDF 27 kb) [file 40462_2018_136_MOESM2_ESM.pdf]

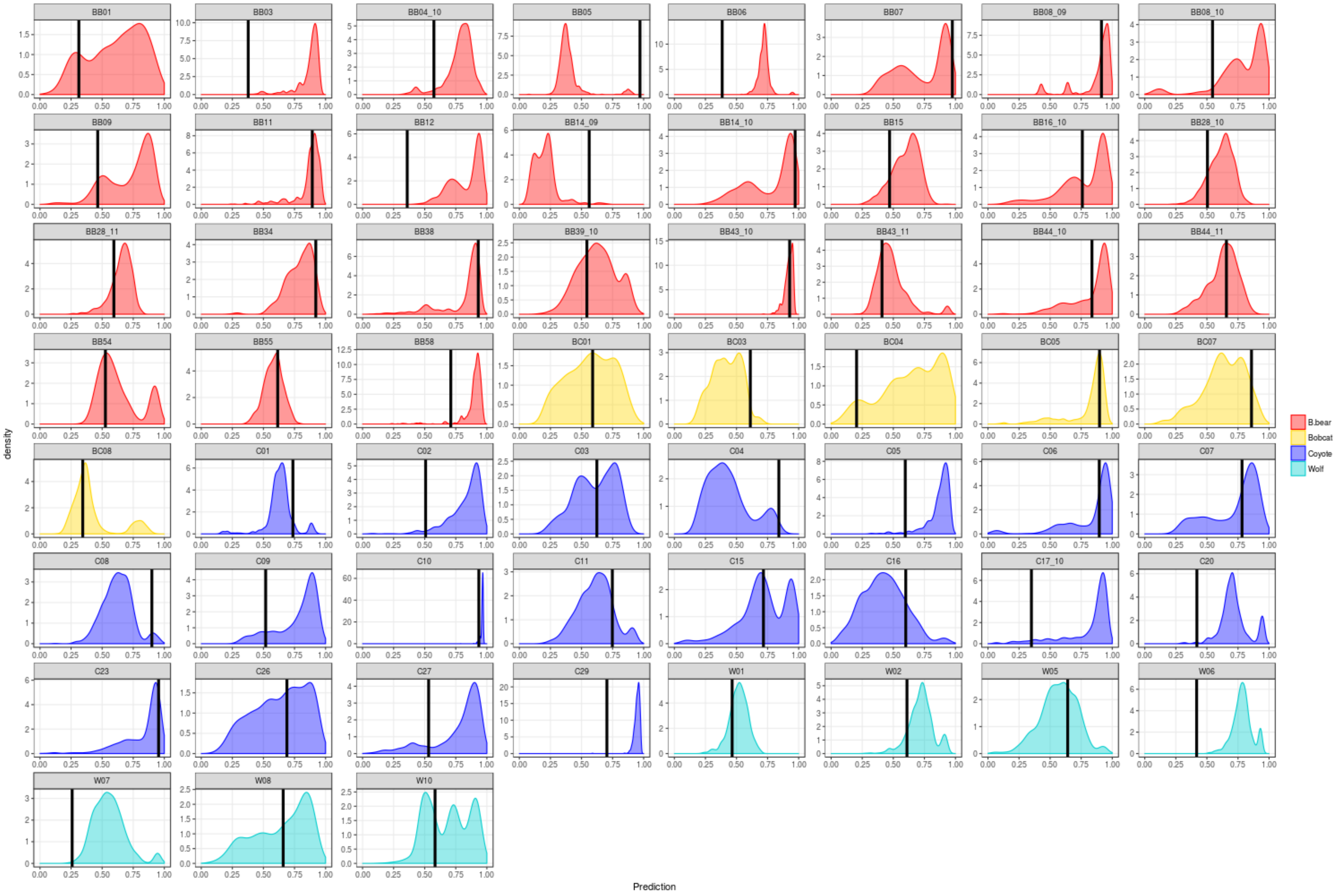

Additional file 10. Comparison between prediction of corridor locations by the *corridor SSF model* and the *non-corridor SSF models*. The black line represents mean prediction value of the *corridor SSF model*, and the colored area represents the distribution of the mean predictions of the 1000 repetitions of the *non-corridor SSF models*. When the line is to the right of the largest peak of the distribution of the predictions of the *non-corridor SSF models*, the *corridor SSF model* could predict better the corridor locations (e.g. BB05, BC07, etc). In all other cases the *non-corridor SSF model* could predict the corridor locations as good or better than the *corridor SSF model*. Red: black bears; yellow: bobcats; dark blue: coyotes; light blue: wolves.
