# Supplementary material for: Habitat suitability does not capture the essence of animal-defined corridors
Source: Mov Ecol. 2018 Sep 27;6:18. doi: 10.1186/s40462-018-0136-2 (PMC6158861; doi:10.1186/s40462-018-0136-2)
Supplement: Supplementary file 3 — Corridor outliers calculation. Left panel: track of one wolf (W01), with all corridors identified by the corridor function of the move R package. Right panel: detail of one section of the track. Corridors A, B and C are considered as outliers. Corridor D would be accepted as corridor. (PDF 110 kb) [file 40462_2018_136_MOESM3_ESM.pdf]

Additional file 2. Environmental variables used

Variables	Description	Source
Land Cover	Water, developed open, developed low, developed medium, developed high, barren, deciduous forest, evergreen forest, mixed forest, shrub, grassland, pasture, crops, woody wetland, herbaceous wetland, rivers, lakes and roads	NLCD11 Classes* Rivers§; Lakes$ Roads#
% Human cover	% of land covers with human presence (urban areas and roads) within a 30 m radius circle from each 30m grid cell	NLCD11 Classes: 21,22,23,24,27* Roads#
% Open cover	% of grasslands, shrubs, crops, barren within a 30 m radius circle from each 30m grid cell	NLCD11 Classes: 31,52,71,81,82,95*
% Evergreen forest	% of evergreen forest within a 30 m radius circle from each 30m grid cell	NLCD11 Class: 42*
% Mixed forest	% of mixed forest within a 30 m radius circle from each 30m grid cell	NLCD11 Class: 43*
% Deciduous forest	% of deciduous forest within a 30 m radius circle from each 30m grid cell	NLCD11 Class: 41*
% Woody wetland	% of woody wetland within a 30 m radius circle from each 30m grid cell	NLCD11 Class: 90*
Distance to roads (m)	Distance from each grid cell to roads	Roads#
Distance to water (m)	Distance from each grid cell to rivers and lakes	NLCD11 Class: 11* Rivers§ ; Lakes$

* Classes correspond to the classification of the National Land Cover Database 2011 legend (Jin et al. 2013 & NLCD11)
# Classification as provided by the United States Census Bureau (US Census Bureau).
§ Rivers correspond to the NHDFlowline layer with the feature type “stream/river” according to the NHD classification (USGS)
$ Lakes correspond to the NHDWaterbody layer, excluding the feature type swamp/marsh (USGS)
