# Supplementary material for: Habitat suitability does not capture the essence of animal-defined corridors
Source: Mov Ecol. 2018 Sep 27;6:18. doi: 10.1186/s40462-018-0136-2 (PMC6158861; doi:10.1186/s40462-018-0136-2)
Supplement: Supplementary file 4 — Detail of corridor polygons and their immediate surrounding areas. (PDF 257 kb) [file 40462_2018_136_MOESM4_ESM.pdf]

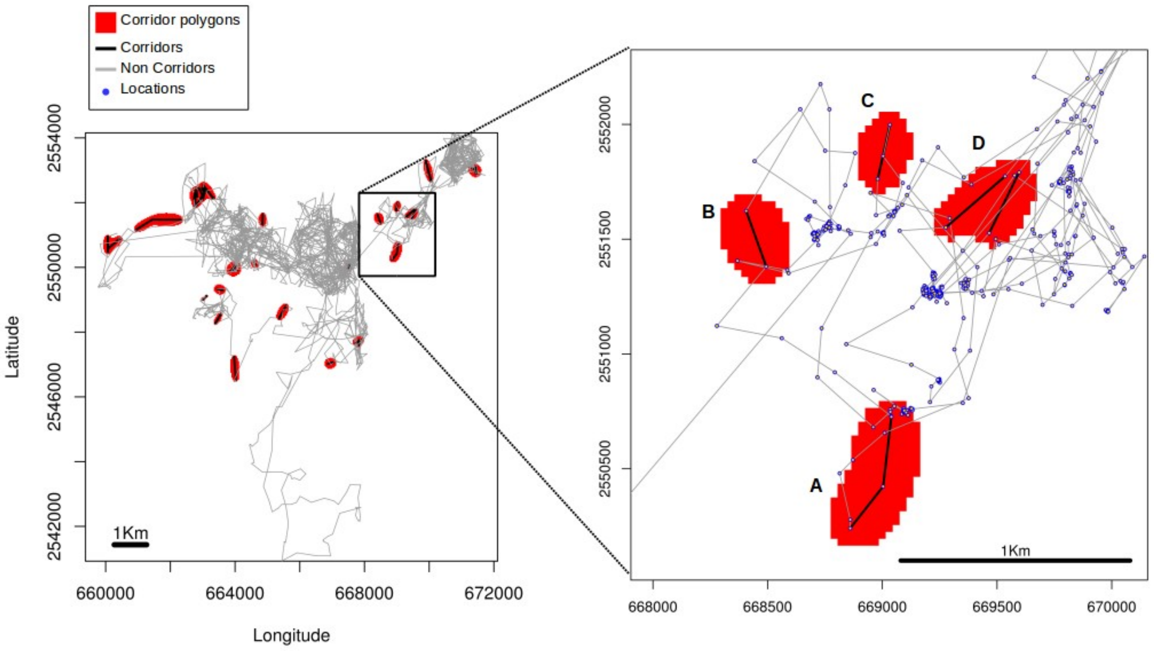

Additional file 3. Corridor outliers calculation. Left panel: track of one wolf (W01), with all corridors identified by the *corridor* function of the *move* R package. Right panel: detail of one section of the track. Corridors A, B and C are considered as outliers. Corridor D would be accepted as corridor.
