# Supplementary figures and images for: Habitat suitability does not capture the essence of animal-defined corridors
Source: Mov Ecol. 2018 Sep 27;6:18. doi: 10.1186/s40462-018-0136-2 (PMC6158861; doi:10.1186/s40462-018-0136-2)

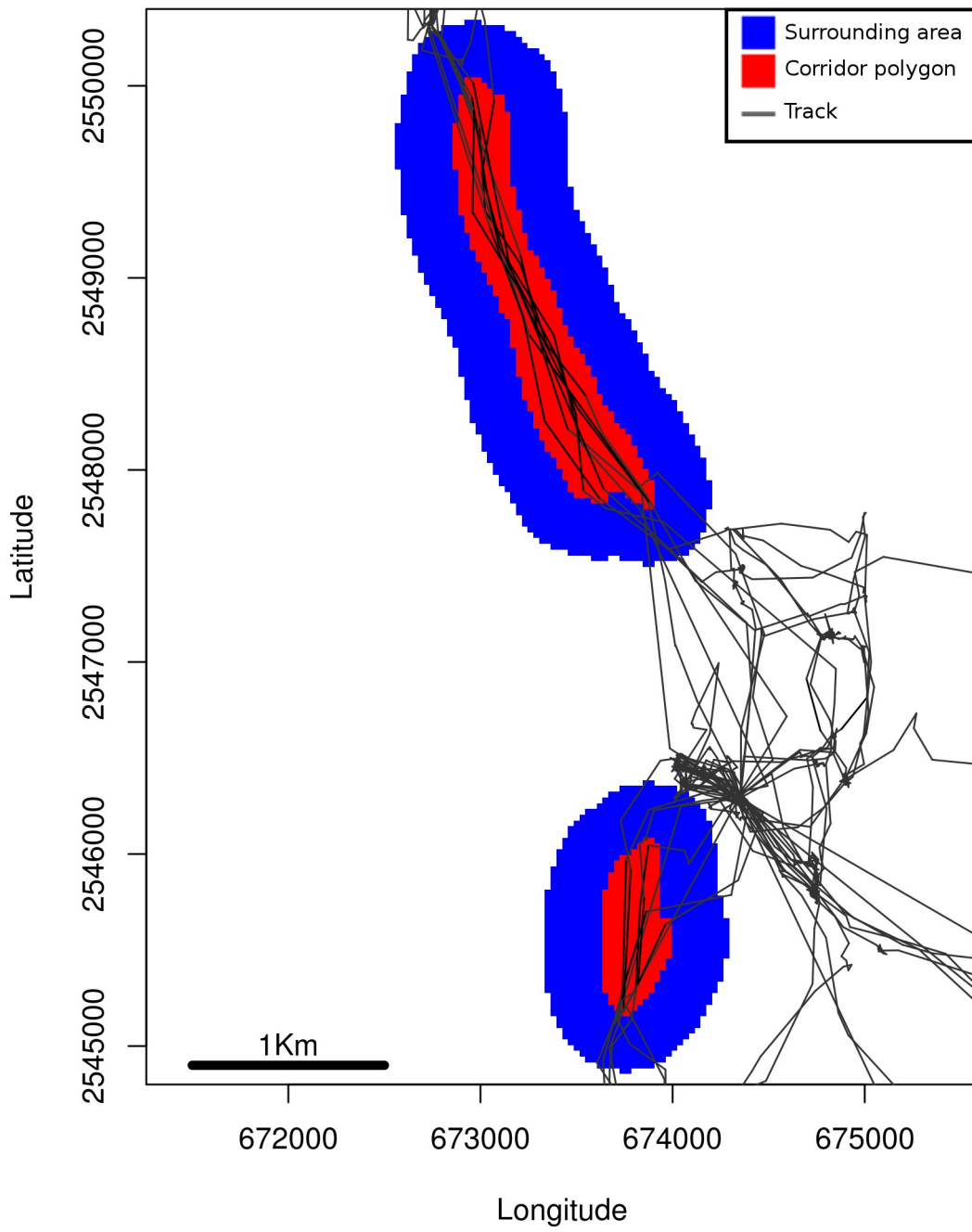

**Additional file 4. Detail of corridor polygons and their immediate surrounding areas.**

Supplement: Supplementary file 5 — Overlap of corridors of the same individual. Percentage of overlap of corridors within one black bear tracked over several years. Each overlapping pair is counted once, always the one with the highest percentage of overlap. “n” represents the number of overlapping pairs of corridors. (PDF 183 kb) [file 40462_2018_136_MOESM5_ESM.pdf]
