# Supplementary material for: Habitat suitability does not capture the essence of animal-defined corridors
Source: Mov Ecol. 2018 Sep 27;6:18. doi: 10.1186/s40462-018-0136-2 (PMC6158861; doi:10.1186/s40462-018-0136-2)
Supplement: Supplementary file 6 — Example of full SSF model and corridor SSF model predictions for one black bear, bobcat, coyote and wolf. Prediction area corresponds to the individuals’ home range. (PDF 4305 kb) [file 40462_2018_136_MOESM6_ESM.pdf]

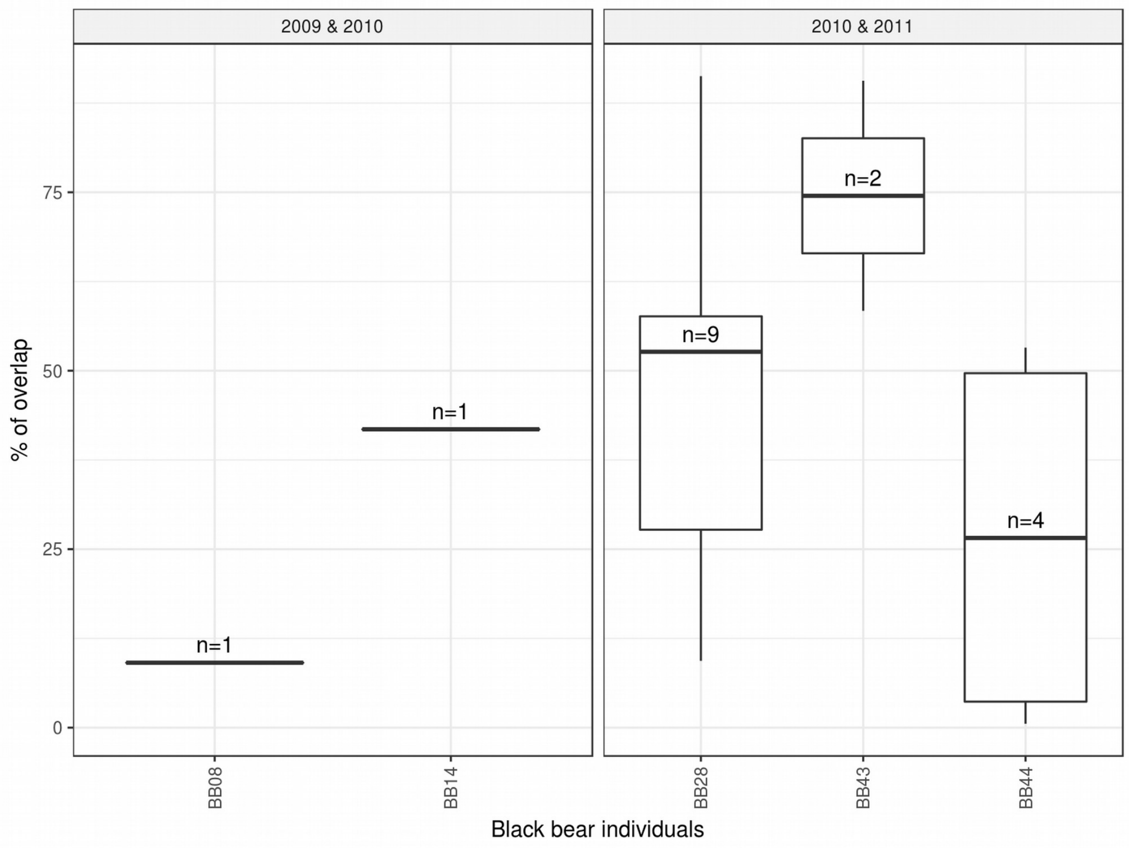

Additional file 5. Overlap of corridors of the same individual. Percentage of overlap of corridors within one black bear tracked over several years. Each overlapping pair is counted once, always the one with the highest percentage of overlap. “n” represents the number of overlapping pairs of corridors.
