# Supplementary material for: Habitat suitability does not capture the essence of animal-defined corridors
Source: Mov Ecol. 2018 Sep 27;6:18. doi: 10.1186/s40462-018-0136-2 (PMC6158861; doi:10.1186/s40462-018-0136-2)
Supplement: Supplementary file 7 — T-test results of the comparison between the habitat suitability value (HS) of corridor vs non-corridor locations from the full SSF model. Negative value of “t” implies that the corridor locations had lower habitat suitability than the non-corridor locations. (PDF 36 kb) [file 40462_2018_136_MOESM7_ESM.pdf]

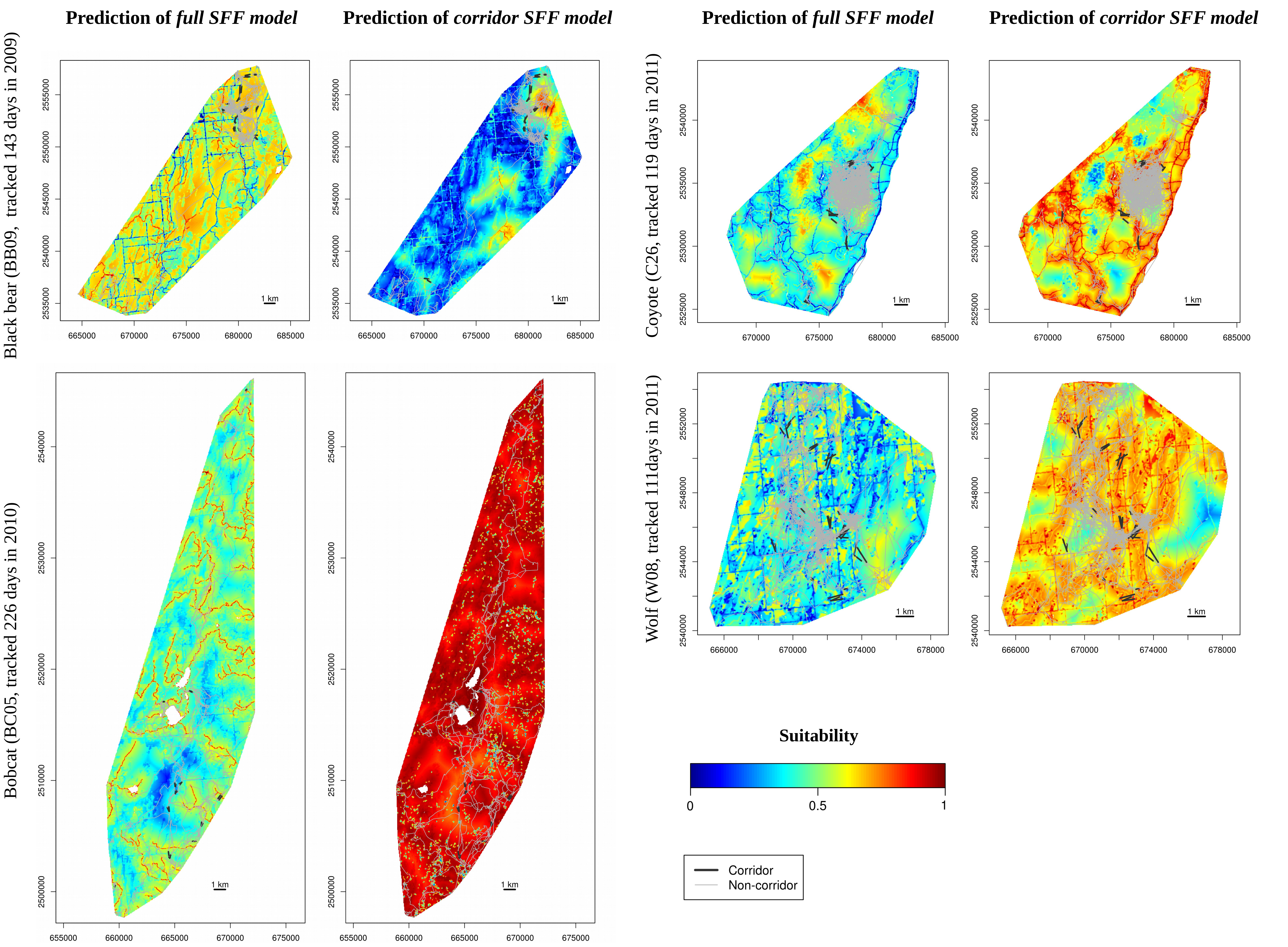

Additional file 6. Example of *full SSF model* and *corridor SSF model* predictions for one black bear, bobcat, coyote and wolf. Prediction area corresponds to the individuals' home range.
