# Supplementary material for: Habitat suitability does not capture the essence of animal-defined corridors
Source: Mov Ecol. 2018 Sep 27;6:18. doi: 10.1186/s40462-018-0136-2 (PMC6158861; doi:10.1186/s40462-018-0136-2)
Supplement: Supplementary file 8 — Paired t-test results of the comparison between the mean habitat suitability value of the corridor polygon and its immediate surrounding area from the full SSF model. Negative value of “t” and “mean of differences” imply that the corridor polygon had lower habitat suitability than the immediate surrounding area. (PDF 33 kb) [file 40462_2018_136_MOESM8_ESM.pdf]

Additional file 7. T-test results of the comparison between the habitat suitability value (HS) of corridor vs non-corridor locations from the *full SSF model.* Negative value of "t" implies that the corridor locations had lower habitat suitability than the non-corridor locations.

**Species**	**Individuals**	**t**	**p-value**	**DF**	**HS corridor (mean±sd)**	**HS non-corridor (mean±sd)**
black bear	BB01	-0.075	0.9399	3559	0.45 ± 0.17	0.45 ± 0.14
black bear	BB03	-1.789	0.0737	7850	0.85 ± 0.08	0.87 ± 0.1
black bear	BB04_09	-4.870	<0.001	8528	0.25 ± 0.1	0.28 ± 0.08
black bear	BB04_10	-4.584	<0.001	2575	0.33 ± 0.09	0.41 ± 0.09
black bear	BB05	-3.463	<0.001	7093	0.42 ± 0.13	0.48 ± 0.14
black bear	BB06	-5.011	<0.001	6713	0.72 ± 0.09	0.76 ± 0.1
black bear	BB07	-7.286	<0.001	5368	0.27 ± 0.11	0.36 ± 0.12
black bear	BB08_09	-4.480	<0.001	7120	0.72 ± 0.07	0.78 ± 0.08
black bear	BB08_10	-5.708	<0.001	13243	0.51 ± 0.1	0.57 ± 0.11
black bear	BB08_11	-2.972	0.003	2455	0.7 ± 0.13	0.77 ± 0.08
black bear	BB09	-8.811	<0.001	6828	0.58 ± 0.1	0.64 ± 0.07
black bear	BB10	-1.090	0.2756	2039	0.72 ± 0.1	0.73 ± 0.06
black bear	BB11	-2.693	0.0071	5801	0.39 ± 0.13	0.44 ± 0.12
black bear	BB12	-2.881	0.004	6029	0.7 ± 0.1	0.73 ± 0.11
black bear	BB14_09	-13.520	<0.001	4914	0.79 ± 0.05	0.92 ± 0.07
black bear	BB14_10	-3.203	0.0014	13059	0.65 ± 0.15	0.7 ± 0.15
black bear	BB15	-5.663	<0.001	10446	0.34 ± 0.11	0.41 ± 0.16
black bear	BB16_10	-9.809	<0.001	13384	0.49 ± 0.1	0.56 ± 0.07
black bear	BB16_11	-0.675	0.5001	1157	0.58 ± 0.13	0.59 ± 0.06
black bear	BB28_10	-7.836	<0.001	13093	0.42 ± 0.14	0.48 ± 0.12
black bear	BB28_11	-14.209	<0.001	12010	0.31 ± 0.13	0.41 ± 0.12
black bear	BB34	0.131	0.8954	7935	0.55 ± 0.07	0.55 ± 0.07
black bear	BB38	-1.073	0.2834	8105	0.51 ± 0.07	0.52 ± 0.08
black bear	BB39_10	-4.945	<0.001	7973	0.36 ± 0.11	0.44 ± 0.12
black bear	BB39_11	-1.450	0.1473	1648	0.23 ± 0.01	0.31 ± 0.12
black bear	BB41	0.138	0.8899	3758	0.51 ± 0.05	0.5 ± 0.08
black bear	BB43_10	-2.116	0.0343	6283	0.45 ± 0.12	0.49 ± 0.12
black bear	BB43_11	-5.518	<0.001	10549	0.52 ± 0.1	0.57 ± 0.1
black bear	BB44_10	-5.098	<0.001	7848	0.34 ± 0.1	0.39 ± 0.1
black bear	BB44_11	-3.425	<0.001	9980	0.35 ± 0.1	0.38 ± 0.09
black bear	BB54	-8.601	<0.001	9224	0.42 ± 0.11	0.47 ± 0.07
black bear	BB55	1.113	0.2658	9547	0.41 ± 0.11	0.4 ± 0.11
black bear	BB58	-0.514	0.6069	6399	0.45 ± 0.08	0.45 ± 0.09
bobcat	BC01	-2.062	0.0392	6574	0.62 ± 0.13	0.67 ± 0.11
bobcat	BC03	-1.492	0.1357	2268	0.37 ± 0.14	0.42 ± 0.13
bobcat	BC04	0.880	0.3789	9034	0.83 ± 0.09	0.82 ± 0.08
bobcat	BC05	-3.237	0.0012	11307	0.48 ± 0.13	0.54 ± 0.14
bobcat	BC06	-1.541	0.1233	3556	0.57 ± 0.04	0.63 ± 0.08
bobcat	BC07	-4.065	<0.001	10894	0.4 ± 0.13	0.49 ± 0.11
bobcat	BC08	-5.623	<0.001	12683	0.67 ± 0.1	0.72 ± 0.09

Species	Individuals	t	p-value	DF	HS corridor (mean±sd)	HS non-corridor (mean±sd)
coyote	C01	-3.373	<0.001	6610	0.56 ± 0.1	0.62 ± 0.11
coyote	C02	-3.579	<0.001	6613	0.56 ± 0.12	0.62 ± 0.09
coyote	C03	-0.939	0.3479	834	0.26 ± 0.12	0.3 ± 0.14
coyote	C04	-3.253	0.0011	8804	0.4 ± 0.13	0.5 ± 0.15
coyote	C05	-2.097	0.0361	8778	0.32 ± 0.12	0.39 ± 0.17
coyote	C06	0.868	0.3853	8631	0.75 ± 0.1	0.74 ± 0.11
coyote	C07	-6.141	<0.001	8512	0.3 ± 0.1	0.42 ± 0.13
coyote	C08	-9.011	<0.001	7874	0.44 ± 0.13	0.55 ± 0.12
coyote	C09	-7.182	<0.001	6788	0.47 ± 0.12	0.57 ± 0.1
coyote	C10	-4.264	<0.001	10613	0.62 ± 0.1	0.67 ± 0.11
coyote	C11	-7.113	<0.001	11073	0.76 ± 0.1	0.81 ± 0.07
coyote	C15	-6.727	<0.001	11791	0.49 ± 0.09	0.55 ± 0.09
coyote	C16	-4.347	<0.001	11102	0.63 ± 0.11	0.68 ± 0.1
coyote	C17_10	-5.536	<0.001	9714	0.41 ± 0.08	0.48 ± 0.1
coyote	C20	-10.774	<0.001	9477	0.73 ± 0.11	0.8 ± 0.07
coyote	C23	-6.118	<0.001	10846	0.61 ± 0.07	0.66 ± 0.06
coyote	C24	-5.534	<0.001	14242	0.4 ± 0.11	0.49 ± 0.11
coyote	C26	-5.568	<0.001	10450	0.36 ± 0.08	0.45 ± 0.1
coyote	C27	-5.203	<0.001	12009	0.63 ± 0.1	0.69 ± 0.07
coyote	C29	-5.210	<0.001	10509	0.43 ± 0.08	0.49 ± 0.09
wolf	W01	-21.002	<0.001	6310	0.55 ± 0.09	0.7 ± 0.11
wolf	W02	-5.123	<0.001	6497	0.44 ± 0.11	0.49 ± 0.08
wolf	W05	-5.735	<0.001	11850	0.42 ± 0.09	0.46 ± 0.08
wolf	W06	-9.216	<0.001	11305	0.24 ± 0.07	0.33 ± 0.12
wolf	W07	-1.553	0.1204	11072	0.41 ± 0.08	0.42 ± 0.09
wolf	W08	-4.157	<0.001	9802	0.4 ± 0.1	0.45 ± 0.11
wolf	W10	-1.755	0.0794	8878	0.46 ± 0.07	0.47 ± 0.06
