# Supplementary material for: Habitat suitability does not capture the essence of animal-defined corridors
Source: Mov Ecol. 2018 Sep 27;6:18. doi: 10.1186/s40462-018-0136-2 (PMC6158861; doi:10.1186/s40462-018-0136-2)
Supplement: Supplementary file 9 — Prediction success of corridor SSF models. Dark gray: individuals where corridor locations had higher prediction value than random locations. Light gray: individuals where random locations had higher prediction value than corridor locations. (PDF 29 kb) [file 40462_2018_136_MOESM9_ESM.pdf]

Additional file 8. Paired t -test results of the comparison between the mean habitat suitability value of the corridor polygon and its immediate surrounding area from the *full SSF model.* Negative value of "t" and "mean of differences" imply that the corridor polygon had lower habitat suitability than the immediate surrounding area.

Species	Individuals	t	p-value	DF	mean of differences
black bear	BB01	0.727	0.500	5	0.0042
black bear	BB03	0.548	0.603	6	0.0078
black bear	BB04_09	-0.887	0.389	15	-0.0063
black bear	BB04_10	-1.795	0.147	4	-0.0303
black bear	BB05	1.756	0.113	9	0.0165
black bear	BB06	0.400	0.695	14	0.0048
black bear	BB07	-0.446	0.664	11	-0.0067
black bear	BB08_09	-0.198	0.850	6	-0.0011
black bear	BB08_10	-0.227	0.824	12	-0.0037
black bear	BB08_11	0.398	0.729	2	0.0159
black bear	BB09	-1.494	0.159	13	-0.0135
black bear	BB10	0.851	0.457	3	0.0152
black bear	BB11	0.990	0.368	5	0.0162
black bear	BB12	0.851	0.412	12	0.0063
black bear	BB14_09	-1.751	0.178	3	-0.0304
black bear	BB14_10	0.563	0.583	13	0.0149
black bear	BB15	0.956	0.347	29	0.0085
black bear	BB16_10	-2.584	0.021	15	-0.0125
black bear	BB28_10	1.939	0.059	41	0.0148
black bear	BB28_11	1.534	0.133	40	0.0126
black bear	BB34	1.932	0.085	9	0.0167
black bear	BB38	-0.746	0.475	9	-0.0061
black bear	BB39_10	-0.399	0.700	8	-0.0051
black bear	BB43_10	0.529	0.616	6	0.0054
black bear	BB43_11	-0.929	0.363	22	-0.0071
black bear	BB44_10	-0.091	0.929	17	-0.0006
black bear	BB44_11	1.974	0.063	19	0.0126
black bear	BB54	-1.684	0.110	17	-0.0204
black bear	BB55	1.214	0.240	18	0.0074
black bear	BB58	1.882	0.102	7	0.0215
bobcat	BC01	1.011	0.387	3	0.0258
bobcat	BC03	-6.260	0.025	2	-0.0362
bobcat	BC04	0.559	0.590	9	0.0054
bobcat	BC05	-0.071	0.945	11	-0.0009
bobcat	BC07	-1.107	0.330	4	-0.0360
bobcat	BC08	0.041	0.968	12	0.0003
coyote	C01	0.301	0.774	6	0.0057
coyote	C02	-1.911	0.129	4	-0.0363

Species	Individuals	t	p-value	DF	mean of differences
coyote	C03	-0.816	0.564	1	-0.0279
coyote	C04	0.346	0.762	2	0.0133
coyote	C05	0.452	0.670	5	0.0057
coyote	C06	-1.073	0.315	8	-0.0107
coyote	C07	1.118	0.314	5	0.0172
coyote	C08	-0.266	0.794	16	-0.0026
coyote	C09	-0.972	0.376	5	-0.0120
coyote	C10	-0.421	0.702	3	-0.0125
coyote	C11	0.721	0.481	16	0.0076
coyote	C15	0.513	0.615	16	0.0057
coyote	C16	0.793	0.445	11	0.0085
coyote	C17_10	1.205	0.256	10	0.0176
coyote	C20	0.112	0.915	5	0.0016
coyote	C23	-0.686	0.542	3	-0.0115
coyote	C24	0.073	0.948	2	0.0011
coyote	C26	-5.398	0.003	5	-0.0193
coyote	C27	-0.912	0.429	3	-0.0187
coyote	C29	0.226	0.827	9	0.0024
wolf	W01	-0.668	0.512	20	-0.0049
wolf	W02	-0.554	0.595	8	-0.0088
wolf	W05	-0.186	0.854	22	-0.0020
wolf	W06	-0.793	0.448	9	-0.0107
wolf	W07	-0.756	0.469	9	-0.0070
wolf	W08	0.177	0.863	10	0.0026
wolf	W10	2.951	0.032	5	0.0388
