# Supplementary material for: Habitat suitability does not capture the essence of animal-defined corridors
Source: Mov Ecol. 2018 Sep 27;6:18. doi: 10.1186/s40462-018-0136-2 (PMC6158861; doi:10.1186/s40462-018-0136-2)
Supplement: Supplementary file 10 — Comparison between prediction of corridor locations by the corridor SSF model and the non-corridor SSF models. The black line represents mean prediction value of the corridor SSF model, and the colored area represents the distribution of the mean predictions of the 1000 repetitions of the non-corridor SSF models. When the line is to the right of the largest peak of the distribution of the predictions of the non-corridor SSF models, the corridor SSF model could predict better the corridor locations (e.g. BB05, BC07, etc). In all other cases the non-corridor SSF model could predict the corridor locations as good or better than the corridor SSF model. Red: black bears; yellow: bobcats; dark blue: coyotes; light blue: wolves. (PDF 246 kb) [file 40462_2018_136_MOESM10_ESM.pdf]

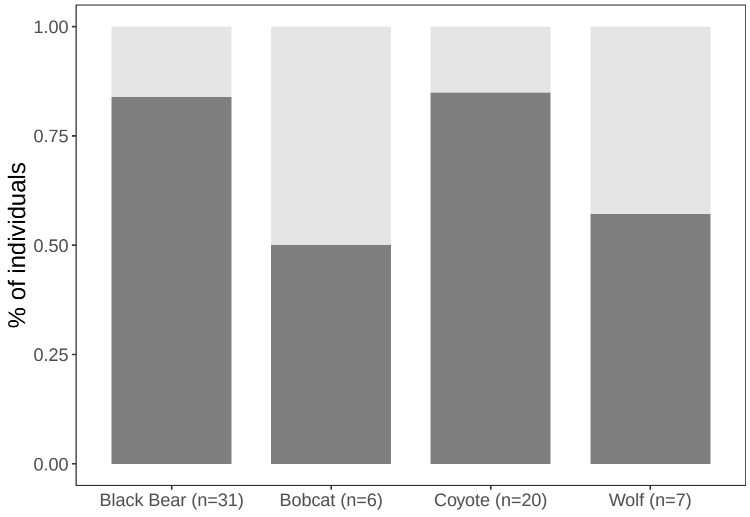

Additional file 9. Prediction success of *corridor SSF models*. Dark gray: individuals where corridor locations had higher prediction value than random locations. Light gray: individuals where random locations had higher prediction value than corridor locations.
